# Development of Biodegradable Nanocarriers Loaded with a Monoclonal Antibody

**DOI:** 10.3390/ijms16023990

**Published:** 2015-02-12

**Authors:** Andrew Gdowski, Amalendu Ranjan, Anindita Mukerjee, Jamboor Vishwanatha

**Affiliations:** 1 Department of Molecular and Medical Genetics, University of North Texas Health Science Center, 3500 Camp Bowie Blvd, Fort Worth, TX 76107, USA; E-Mails: andrewgdowski@gmail.com (A.G.); Amalendu.Ranjan@unthsc.edu (A.R.); Anindita.Mukerjee@unthsc.edu (A.M.); 2 Texas College of Osteopathic Medicine, University of North Texas Health Science Center, 3500 Camp Bowie Blvd, Fort Worth, TX 76107, USA; 3 Institute of Cancer Research, University of North Texas Health Science Center, 3500 Camp Bowie Blvd, Fort Worth, TX 76107, USA

**Keywords:** antibody, nanoparticle, cancer, PLGA, therapy, formulation

## Abstract

Treatments utilizing monoclonal antibody therapeutics against intracellular protein-protein interactions in cancer cells have been hampered by several factors, including poor intracellular uptake and rapid lysosomal degradation. Our current work examines the feasibility of encapsulating monoclonal antibodies within poly(lactic-*co*-glycolic acid) (PLGA) nanoparticles using a water/oil/water double emulsion solvent evaporation technique. This method can be used to prepare protective polymeric nanoparticles for transporting functional antibodies to the cytoplasmic compartment of cancer cells. Nanoparticles were formulated and then characterized using a number of physical and biological parameters. The average nanoparticle size ranged from 221 to 252 nm with a low polydispersity index. Encapsulation efficiency of 16%–22% and antibody loading of 0.3%–1.12% were observed. The antibody molecules were released from the nanoparticles in a sustained manner and upon release maintained functionality. Our studies achieved successful formulation of antibody loaded polymeric nanoparticles, thus indicating that a PLGA-based antibody nanoformulation is a promising intracellular delivery vehicle for a large number of new intracellular antibody targets in cancer cells.

## 1. Introduction

Approximately 30 monoclonal antibody therapeutics have been approved for clinical use in the United States and more are in various stages of clinical trials [1]. Research and treatments with antibodies used for cancer therapy have been limited to targeting extracellular or secreted antigens because in the infrequent event that non-receptor medicated endocytosis of an antibody occurs, the antibody will be destined for the harsh environment of the lysosome and thus rendered inactive [2]. One strategy to overcome this obstacle and achieve intracellular delivery of antibodies is to encapsulate the antibody molecules within polymeric nanoparticles. The nanoparticles can protect the antibody while in transit through the circulation and when the nanoparticles are endocytosed by the cancer cells they have the ability to rapidly escape the lysosomal compartment [3], degrade, and release the antibody molecules inside the cancer cell’s cytoplasmic compartment.

Poly(lactic-*co*-glycolic acid) (PLGA) is a biodegradable polymer that has been approved by the US Food and Drug Administration [4]. Numerous examples exist in the literature demonstrating PLGA as a controlled release nanoparticle vehicle for various drug molecules including: small hydrophobic molecules [5], nucleic acids [6], and proteins [7]. In addition, many groups have used antibodies on the outside of the nanoparticles for targeting purposes. However, to our knowledge no studies have reported using antibodies encapsulated inside PLGA nanoparticles for cancer treatment and research.

For characterization purposes we have chosen to use an antibody against AnnexinA2 (AnxA2) as a model antibody in this formulation. AnxA2 is a calcium-dependent phospholipid binding protein found on various cell types. AnxA2 is highly expressed in certain cancer cells and plays multiple roles in regulating cellular functions including: angiogenesis, proliferation, apoptosis, cell migration, invasion, and adhesion [8]. Anti-AnxA2 antibody was chosen because its functionality can be validated in both western blots and immunofluorescence experiments.

In this article, we present evidence that monoclonal antibodies can be successfully encapsulated using a modified water in oil in water (w/o/w) double emulsion solvent evaporation technique. Further, we demonstrate that when using this method of antibody encapsulation, target binding of the released antibody is maintained. This strategy for delivering functional antibodies inside cancer cells has the potential to open up a large number of new targets for antibody based therapeutics.

## 2. Results and Discussion

**Figure 1:**
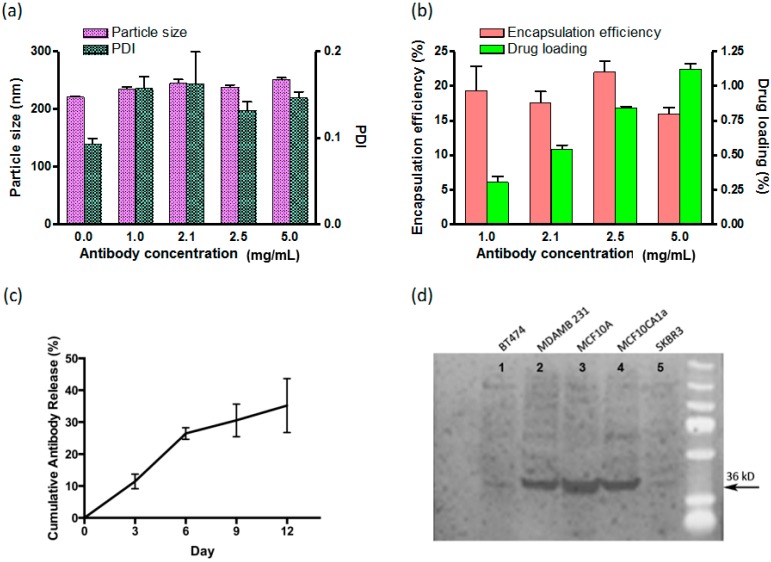
Characterization of anti-Anx A2 antibodies encapsulated within poly(lactic-*co*-glycolic acid) (PLGA) nanoparticles. (**a**) Dynamic Light Scattering (DLS) measurement of the size and polydispersity index (PDI) of the nanoparticles; (**b**) Encapsulation efficiency and drug loading of the nanoparticles; (**c**) Twelve day cumulative antibody release experiment; and (**d**) Immunoblot of whole cell lysates from breast cancer cell lines showing functional binding of released AnnexinA2 (AnxA2) antibody from the nanoparticle at 36 kD. The BT474 and SKBR3 cell lines are known to have very low AnxA2 expression. MDAMB231, MCF10A, and MCF10CA1a cell lines are known to have high AnxA2 expression. (Bars represent standard error of the mean, *n* = 3).

The double emulsion with solvent evaporation technique yielded antibody encapsulation efficiency ranging from 16% to 22% (Figure 1). This represents an adequate value given the large dimensions of IgG molecules (14.2 nm × 8.5 nm × 3.8 nm) [9] and hydrophilic nature of antibodies. Higher drug loading can be achieved if nanoparticle size is increased, however the goal of this formulation was to create nanoparticles that would be able to utilize the enhanced permeability and retention effect for delivery to solid tumors [10]. Thus, the hydrodynamic diameter was limited to less than ~250 nm in all formulations (Figure 1). As expected, the hydrodynamic size of the nanoparticles tended to slightly increase as we increased the initial antibody concentration and more antibody molecules were loaded into the nanoparticles (Figure 1).

Release kinetics performed over the course of 12 days revealed the antibodies were released from the nanoparticles in a sustained manner (Figure 1c).

**Figure 2:**
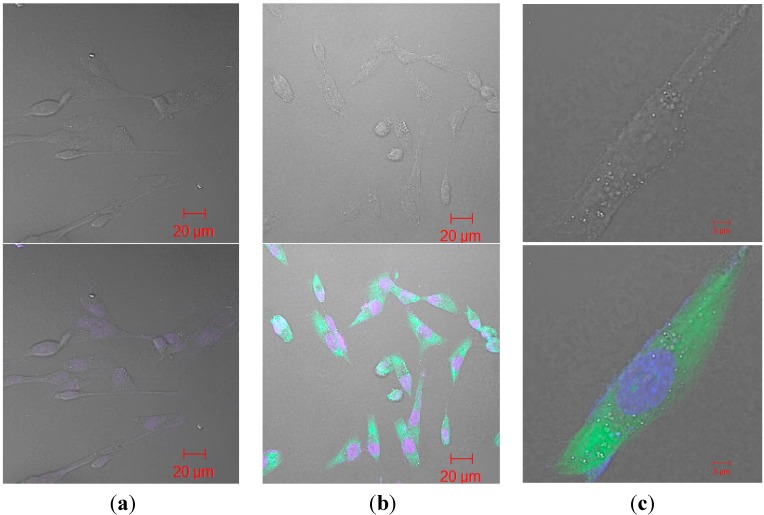
Immunofluorescence of released anti-AnxA2 antibody from nanoparticle (**a**) MDAMB 231 cells treated with heat inactivated anti-AnxA2 antibody released from nanoparticle; (**b**) Lower magnification of MDAMB 231 cells treated with anti-AnxA2 antibody released from nanoparticles; (**c**) Higher magnification of single MDAMB 231 cell treated with released anti-AnxA2 antibody. Green = Alexa fluor 488 tagged secondary antibody. Blue = DAPI.

Finally, antibody functionality was assessed after release from the nanoparticles to demonstrate that in fact binding specificity of the antibody remained intact after undergoing the chemical and physical stresses during the encapsulation process. The released antibody was able to show a strong signal when binding to the whole cell lysates from cell lines of high AnxA2 expression while minimal signal was detected on low AnxA2 cell lines (Figure 1). In addition, immunofluorescence staining using the released antibody also showed maintained antibody functionality (Figure 2).

## 3. Materials and Methods 

Anti-AnxA2 encapsulated PLGA nanoparticles (AbNPs) were prepared using a double emulsion with solvent evaporation technique. Mouse monoclonal anti-AnxA2 (D1/274.5) antibody was a kind gift from Dr. Tony Hunter, Salk Institute for Biological Studies, La Jolla, CA, USA. D1/274.5 was generated from hybridoma cells and are isotype IgG2a. Encapsulation was performed with AnxA2 antibody (D1/274.5) used at various initial concentrations (0, 1, 2.1, 2.5, 5 mg/mL) by diluting in PBS pH 7.4, added into 2 mL PLGA (50:50) ethyl acetate solution, mixture was vortexed for 30 s, then sonicated on ice at 40% continuous intensity for two 30 s time periods with a ten second break in between. Primary emulsion was transferred into 10 mL of 2% poly(vinyl alcohol) (PVA) and sonicated on ice at 40% intensity on intermittent setting for 1 min. Organic solvent was evaporated at atmospheric pressure by magnetic stirring. Next, nanoparticles were washed three times by centrifuging three times at 18,000× *g* for 40 min and washed with water at the end of each centrifugation time point. The nanoparticles were resuspended on the final wash, flash frozen, and lyophilized. The nanoparticles were stored at 4 °C for further use.

Nanoparticles were characterized to determine hydrodynamic particle size and polydispersity index (PDI) by dynamic light scattering using the Zetasizer Nano ZS instrument (Malvern Ltd., Worcestershire, UK).

Encapsulation efficiency was determined by setting up a standard curve of known anti-AnxA2 antibody in Bis-Tris polyacrylamide gels. Samples were prepared by addition of 2 mg AbNP into 5% 2-mercaptoethanol (BME) reducing dye, boiled, and loaded into the gel. Coomassie Brillant Blue R-250 staining (Thermo Fisher Scientific Inc., Rockford, IL, USA) was used for quantification using linear regression calculations based on Image J analysis of heavy chains.

*In vitro* release kinetics were carried out by addition of 2 mg of AbNP to 1 mL of PBS solution pH 7.4. Nanoparticles were continuously mixed at 37 °C. At specified time points, tubes were centrifuged at 18,000 × *g* for 40 min to pellet the nanoparticles. Supernatant with released antibody were collected and quantified by bicinchoninic acid (BCA) kit (Thermo Fisher Scientific Inc., Rockford, IL, USA).

Antibody functionality after release from nanoparticles was determined by adding the AbNP to 1 mL of PBS pH 7.4 and continuously mixed for 9 days at 4 °C. Nanoparticles were then pelleted by centrifugation at 18,000× *g* for 40 min, supernatant was collected and released antibody was used as the primary antibody for western blot detection of AnxA2 in various breast cancer whole cell lysates.

Confocal images were obtained using LSM 510 confocal microscope (Zeiss, Pleasanton, CA, USA) after human breast cancer cells (MDA-MB-231) were grown on coverslips, permeabilized, fixed, treated with anti-AnxA2 that was released from nanoparticles, and labeled with Alexa Fluor 488 goat anti-mouse IgG secondary antibody (Sigma Aldrich, Saint Louis, MO, USA).

## 4. Conclusions

This study provides evidence for utilizing PLGA nanoparticles as sustained release vehicles for the intracellular delivery of therapeutic antibodies to cancer cells. Delivery of functional antibodies to the cytoplasmic compartment may enable these antibodies to target numerous aberrant intracellular biomolecules for cancer treatment. Furthermore, this method of antibody delivery might also have utility studying various biological processes in the context of live intracellular imaging with fluorescently labeled antibodies.
